# Alterations of sleep quality and circadian rhythm genes expression in elderly thyroid nodule patients and risks associated with thyroid malignancy

**DOI:** 10.1038/s41598-021-93106-x

**Published:** 2021-07-01

**Authors:** Xudan Lou, Haidong Wang, Yanyuan Tu, Wen Tan, Cuiping Jiang, Jiao Sun, Zhijun Bao

**Affiliations:** 1 Department of Endocrinology, Huadong Hospital Affiliated to Fudan University, Shanghai, 200040 People’s Republic of China; 2 The Shanghai Key Laboratory of Clinical Geriatric Medicine, Shanghai, 200040 People’s Republic of China; 3 Department of General Surgery, Huadong Hospital Affiliated to Fudan University, Shanghai, 200040 People’s Republic of China; 4 Department of Gastroenterology, Huadong Hospital Affiliated to Fudan University, Shanghai, 200040 People’s Republic of China; 5 Research Center On Aging and Medicine, Fudan University, Shanghai, 200040 People’s Republic of China; 6 Department of Geriatrics, Huadong Hospital Affiliated to Fudan University, Shanghai, 200040 People’s Republic of China

**Keywords:** Thyroid diseases, Risk factors, Thyroid cancer, Gene expression analysis

## Abstract

To study the alterations of sleep quality and circadian rhythm genes expressions upon elderly thyroid nodule patients, the risk factors associated with thyroid malignancies, and the potential relationship involved. The elderly people enrolled in our study were divided into three groups according to the thyroid histopathology: malignant nodule group, benign nodule group, and normal group, and the clinical data and sleep quality were collected. Among the patients of surgery, 56 fresh thyroid tissues were collected for real-time PCR, immunohistochemistry and western blotting analysis of CLOCK, BMAL1, CRYs and PERs. Poor sleep quality, sleep latency and daytime dysfunction were the independent risk factors of malignant nodule after adjusted by other impacts. The expression levels of CLOCK, BMAL1 and PER2 in thyroid malignant group were significantly higher than benign and normal groups, while CRY2 was decreased, *p* < 0.05. In addition, CLOCK and BMAL1 protein levels were positively correlated with PSQI of corresponding patients and CRY2 was negatively correlated. Circadian rhythm genes mainly altered in malignant nodules, and sleep disorders may be involved in the occurrence of elderly thyroid malignancy through the high expressions of CLOCK and BMAL1, and low expression of CRY2.

## Introduction

Thyroid cancer is the most common endocrine malignancy, accounting for 1% of the whole human cancers^1^. According to an AACE/ACE disease state clinical review, the incidence of thyroid cancer has been relatively stable in the past 30 years, and the growth rate began to grow rapidly in 1990s^2^. With the development of aging society, those aged 65 years and over have been the largest burden of the growth, within the incidence increased from 8 to 23.4 per 100,000 population in 2011^2^. The high incidence of cancer in the elderly has brought huge pressure and burden to social pension, medical service and family economy. It is worth noting that an epidemiological study of postmenopausal non obese women showed that insomnia was associated with a high incidence rate of thyroid cancer^3^. Therefore, the adverse health consequences of sleep interruption and sleep duration shortening have caused widespread concerns and arguments, for the two phenomena are prevalent in modern society.

Circadian rhythms or circadian clocks are periodic phenomenon, driven by a series of transcription and translation loops, post-translational modification and degradation mechanisms of clock genes, which make the physiology, biochemistry, behavior and other life activities of the body showing an amplitude oscillation with an approximately 24 h cycle^4^. The circadian transcriptional and translational feedback loop is mainly mediated by various activators, such as CLOCK (circadian locomotor output cycles kaput), BMAL1 (brain and muscle Arnt-like protein-1), and their target genes, PER1, PER2, CRY1, and CRY2, which constitute negative repressor complexes that interact with CLOCK and BMAL1 to inhibit PER and CRY gene transcription^5^. The mutation of biological clock genes not only destroy the normal function of circadian rhythms, but also have been linked to cell cycles disorder, cell proliferation, DNA damage, and apoptosis control, which are closely related to many kinds of human cancers^6,7^. So far, many people have not realized that the interruption of human body's biological clock may play an important role in human health, including cancer. For instance, perturbation of circadian rhythms both in humans (shift workers) and animals has been associated with malignant transformations^8^. This is a new field, the complexity of the regulation of peripheral clock is just beginning to be explored and recognized, which deserves our attention.

Every cell and organ in the human body has a biological clock connected with the brain center clock, suprachiasmatic nucleus (SCN). Turning to the thyroid gland regulating hormones, both TRH and TSH exhibit pronounced circadian oscillations in the blood with a peak between 2:00am and 4:00am in healthy subjects^9^. Moreover, low-amplitude circadian variations were reported for the thyroid hormones total T_3_ and T_4_, suggesting circadian function for the thyroid gland^10,11^. In addition, the rate of thyroid cancer was significantly increased when TSH secretion disorders, lower levels of TSH were associated with a lower risk of papillary thyroid cancer in patients with thyroid nodular disease^12^, indicating that the biological rhythm might be related to the occurrence of thyroid cancer.

Of note, studies have shown that circadian clock genes were associated with sleep outcomes in human research^13^. Thus, the essential question we addressed in our study was whether sleep quality and circadian rhythm genes expression were altered upon thyroid nodule patients, especially malignant nodules; whether poor sleep quality was the risk factor associated with thyroid malignancies; and whether there was a correlation between sleep disorders and circadian rhythm genes, which may be involved in the development of thyroid malignancy. We mainly focus on elderly people because of their own sleep characteristics and high rate of thyroid cancer. From the clinical perspective, the findings of alterations of sleep quality and expression levels of core clock genes in thyroid nodule patients might help improve thyroid malignancies preoperative diagnostics and provide new ideas for clinical treatments.

## Materials and methods

### Study of the subjects

**Figure 1:**
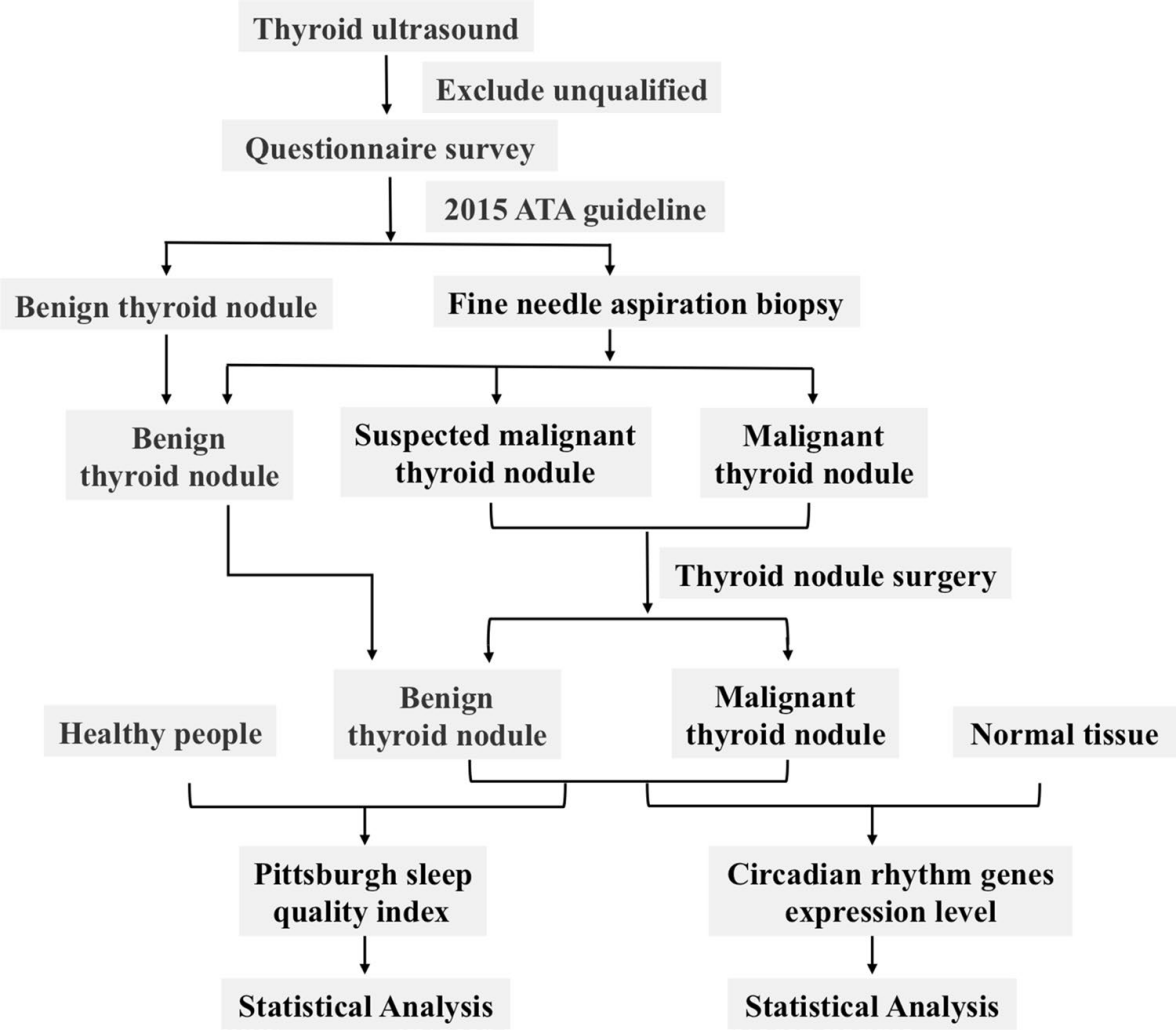
The whole processes of our study.

The process of the whole study was shown in Fig. 1. Thyroid nodules patients were collected from January 2019 to December 2020 in the Department of Endocrinology, Huadong Hospital, Shanghai, China. Based on the inclusion and exclusion criteria, eligible patients were enrolled. Thyroid fine-needle biopsy was performed according to 2015 American Thyroid Association (ATA) guideline^14^. Then combined with biopsy pathology, these patients were divided into thyroid benign nodule group and thyroid malignant group. Patients with suspected malignant nodules underwent surgical resection and pathological diagnosis. Meanwhile, healthy people of similar age and diagnosed with routine physical examination were selected in normal control. All the participants needed to complete the Pittsburgh sleep quality scale questionnaire survey, and informed consent was obtained from all subjects. The clinical study was approved by the ethics committee of Huadong Hospital Affiliated to Fudan University and all the subjects were registered anonymously.

### Inclusion and exclusion criteria

Inclusion criteria: patients with thyroid nodule were diagnosed as thyroid nodule according to the DTC treatment guidelines of thyroid nodule and differentiated thyroid cancer in the American Thyroid Association in 2015^14^. Age: ≧60 years old; conscious and able to communicate normally; signed informed consent.

Exclusion criteria: exclusion of patients with cancer, cardiovascular and cerebrovascular diseases, liver and kidney dysfunction; exclusion of obstructive sleep apnea syndrome; exclusion of patients with diabetes, irritable bowel syndrome, serious mental illness; exclusion of other thyroid diseases and patients refusing to cooperate.

According to the pathological results after thyroid fine needle aspiration and operation, the patients were divided into two groups.

### Sleep quality

Pittsburgh sleep quality Index (PSQI) is a self-reported questionnaire that assesses sleep quality and disorders. It contains seven component scores: subjective sleep quality, sleep latency, sleep duration, habitual sleep efficiency, sleep disturbance, use of sleeping medication, and daytime dysfunction^15^. The score range of each question is 0–3, and the total score is 0–21. The higher of the score, the worse of the sleep quality. The sum of the scores for these seven components determined sleep quality classification: more than 7 points indicated poor sleep quality, and less than or equal to 7 points indicated good sleep quality (the threshold of poor sleep quality was 7 points in Chinese people as reported^16^). All participants had 15 min to complete the questionnaire under the guidance of one trained researcher.

### Thyroid tissue sample

Fresh thyroid tissue samples (0.5–1cm^3^) were obtained from patients undergoing thyroidectomy for thyroid cancer or suspicious nodules, with the written informed consent. The biopsy material was collected in a daytime dependent manner, with all the surgeries performed in the time window between 8:00 AM and 2:00 PM. Malignant tumors were classified by histopathological analysis according to the World Health Organization Histological Classification of Thyroid Tumors^17^. In order to avoid the abnormal biochemistry or immunity of the tissues adjacent to the malignancy, we selected the normal tissues more than 5 mm away from the nodules. The obtained thyroid tissue was deep frozen and kept for next analysis. The study protocol was approved by the local ethics committee as mentioned above.

### Immunohistochemistry analysis

The thyroid tissues were processed for paraffin embedding, sliced into 2-to 3-µm-thick cryosections, and adhered to Super-frost Plus slides. All the sections were stained using a SuperPolymer rabbit and mouse horseradish peroxidase (HRP) kit (CoWin Bioscience Co. Ltd, Beijing, China) as previously described^18^. After xylene dewaxing, gradient ethanol hydration, and antigen retrieval in citrate buffer, the tissues were washed three times with 0.01 M phosphate-buffered saline (PBS), pH 7.4, and incubated with primary antibodies (Cell Signaling Technology, Massachusetts, USA) for 1 h at 37 °C. The secondary antibody with the streptavidin–biotin–HRP complex (Bioss Inc., Massachusetts, USA) was then added to the system and incubated for 30 min at 37 °C. After coloration with 3,3-diaminobenzi-dine, the slides were counterstained with hematoxylin, mounted, and visualized using light microscopy.

### Real-time qPCR assay

The total RNA was isolated from thyroid tissues with Trizol reagent (Invitrogen Life Technologies Inc., Carlsbad, CA, USA), GAPDH was used as internal control. RNA was finally dissolved in 0.1% diethyl pyrocarbonate (DEPC). The content of RNA was determined from the ratio of optical density (OD260/280), while the quality was analysed by agarose gel electrophoresis. A quantity of 1 μg RNA was reverse-transcribed using MMLV reverse transcription system (Promega, Madison, WI, USA) as previously described^19^. The cDNA was then used in the PCR amplification system. Stained with ethidium bromide, the PCR products and another 100 bp DNA ladder were run in a 2% agarose gel electrophoresis to detect if the PCR products had only one specific amplified band. Real-time PCR was performed by the ABI Prism 7000 Sequence Detection System (Applied Biosystems, Foster City, CA, USA) using SYBR Green fluorescence signals, and melting curves were obtained to analyze the specificity of amplification and to check the contamination of genomic DNA. The cDNA PCR products were tenfold gradient diluted, from 1 × 10^−1^ to 1 × 10^−9^, real-time PCR was performed by ABI Prism 7300 SDS Software to obtain the standard curve, where Ct was the threshold value. Relative expression of mRNA was calculated after normalization to GAPDH, using the comparative 2^−ΔΔCt^ method as described previously^20^.

### Western-blotting

The protein concentrations of the tissue extracts (30 μg) were determined using the bicinchoninic acid (BCA) assay system, according to the company’s Manual (Uptima; Interchim, Montlucon, France) as previously described^18^. 500 ng proteins per lane were separated by sodium dodecyl sulfate polyacrylamide gel electrophoresis (SDS-PAGE, 5% for stacking gel and 8% for separating gel) and then transferred onto nitrocellulose membranes. The membranes containing the proteins were pre-incubated in blocking buffer (5% (w/v) skimmed milk powder in PBS/0.1% Tween 20) for 1 h, and then incubated with primary antibodies against CLOCK, BMAL1, CRY1, CRY2, PER1, PER2 (Cell Signaling Technology, Massachusetts, USA) and β-actin (overnight, 4 °C). After being washed three times with blocking buffer, the membranes were incubated with alkaline phosphatase-conjugated secondary antibodies (GeneTex, Southern California, USA) for 120 min and were finally washed in 0.1 M Tris, pH 9.5. The density (specific binding) of each band was measured by densitometry using Quantity One (Bio-Rad Laboratories Inc., Munich, Germany).

### Statistical analysis

All results were expressed as means ± standard deviations. Analysis of comparisons between groups was performed by Student’s T-test or one-way ANOVA after normality assessment. Categorical variables were analyzed by using the chi-squared test. Pearson’s correlation coefficient was calculated to determine statistical relationships between variables. Logistic regression statistical analyses were carried out using SPSS 23.0 software for Mac. The figures were drawing with Image J and GraphPad Prism 8. A level of probability up at *p* < 0.05 was set up as statistically significant.

### Ethical approval

The study was carried out in accordance with The Code of Ethics of the World Medical Association (Declaration of Helsinki) for experiments involving humans, and approved by the Ethics Committee of our institute.

## Results

### Participant characteristics

We totally collected 580 thyroid nodule patients aged from 60 to 86 years by B ultrasound. According to 2015 ATA guideline as well as in- and ex-criteria, 154 subjects were excluded. Through fine needle aspiration biopsy and surgery of thyroid, the patients were divided into benign nodule group (n = 354) and malignant nodule group (n = 72). At the same time, 53 healthy elderly people were in another group as control. The participants are all from Shanghai, China, where the average urine iodine median was 138.4 µg/L^21^. Among the participants, none of them had autoimmune diseases but a history of radiation exposure.

The characteristics of the study subjects were shown in Table 1. Malignant group scored higher on sleep duration, sleep disturbance, daytime dysfunction as well as PSQI when compared with benign group, *p* < 0.05. Similarly, we found that malignant people performed worse on sleep latency, sleep duration, daytime dysfunction and PSQI than normal group subjects, *p* < 0.05. Thyroid autoantibodies (TGAB, TPOAB, TRAB) were statistically significant in the three groups, while TSH was no difference.

**Table 1:** Clinical characteristics and sleep quality of participants in thyroid malignant group, benign nodule group and normal group.

	Malignant	Benign	Normal	*P*_*1*_ value	*P*_*2*_ value
Number (M/F)	72(22/50)	354(74/280)	53(44/9)	0.117	0.000
Age (years)	67.26 ± 5.25	68.23 ± 6.36	67.20 ± 5.24	0.175	0.953
Duration (years)	2.95 ± 3.96	3.64 ± 4.98	N/A	0.199	N/A
BMI (kg/m2)	25.55 ± 5.54	24.19 ± 8.15	25.12 ± 3.93	0.119	0.633
Systolic BP (mmHg)	120.40 ± 19.59	122.00 ± 12.68	119.66 ± 16.45	0.551	0.108
Diastolic BP (mmHg)	82.27 ± 8.60	74.54 ± 6.98	78.18 ± 8.11	0.000	0.011
Cholesterol (mmol/L)	4.75 ± 1.02	5.87 ± 3.36	4.52 ± 0.99	0.235	0.276
Triglyceride (mmol/L)	1.50 ± 1.07	2.57 ± 2.52	1.95 ± 1.23	0.287	0.058
LDL-C (mmol/L)	1.87 ± 0.71	3.02 ± 1.02	2.67 ± 0.82	0.273	0.339
HDL-C (mmol/L)	1.44 ± 0.29	1.68 ± 0.37	1.16 ± 0.22	0.521	0.215
HbA1c(%)	6.20 ± 0.87	6.12 ± 2.78	5.91 ± 0.69	0.213	0.629
FBG (mmol/L)	5.39 ± 1.42	5.69 ± 1.16	5.58 ± 0.70	0.170	0.362
UA (umol/L)	309.18 ± 90.89	297.22 ± 79.16	353.85 ± 100.23	0.832	0.014
FT3 (pmol/L)	4.21 ± 0.69	4.35 ± 3.30	3.89 ± 0.54	0.474	0.007
FT4 (pmol/L)	12.91 ± 2.06	12.96 ± 1.82	13.09 ± 1.55	0.838	0.593
TSH (mIU/L)	2.28 ± 5.60	2.07 ± 2.62	2.05 ± 1.90	0.636	0.962
TGAB (IU/ml)	80.85 ± 230.72	24.33 ± 85.26	2.11 ± 4.07	0.001	0.059
TPOAB (IU/ml)	137.62 ± 305.33	48.66 ± 177.21	0.52 ± 1.15	0.003	0.049
TRAB (IU/ml)	34.29 ± 104.16	21.01 ± 36.92	0.50 ± 0.61	0.088	0.000
TG (ng/ml)	49.04 ± 154.22	53.81 ± 173.28	13.04 ± 10.63	0.854	0.139
SAS score	37.06 ± 7.68	36.63 ± 6.74	36.22 ± 7.72	0.658	0.125
SDS score	33.77 ± 6.28	30.20 ± 6.61	34.20 ± 7.65	0.000	0.692
Sleep quality score	1.43 ± 0.60	1.40 ± 0.79	1.21 ± 0.74	0.722	0.076
Sleep latency score	1.29 ± 0.84	1.09 ± 1.07	0.60 ± 0.59	0.091	0.000
Sleep duration score	1.43 ± 0.78	1.21 ± 0.88	1.15 ± 0.69	0.037	0.037
Sleep efficiency score	1.33 ± 0.82	1.15 ± 1.11	1.13 ± 0.65	0.114	0.130
Sleep disturbance score	1.48 ± 0.71	1.27 ± 0.59	1.39 ± 0.63	0.020	0.458
Sleeping medication score	0.48 ± 0.76	0.41 ± 0.87	0.39 ± 0.66	0.437	0.485
Daytime dysfunction score	1.73 ± 0.67	1.46 ± 1.11	0.75 ± 0.67	0.007	0.000
PSQI score	9.19 ± 2.59	8.01 ± 3.89	6.64 ± 2.71	0.002	0.000

Data expressed as means ± SD or number. *P*_1_ value was a comparison between thyroid malignant and benign groups. *P*_2_ value was a comparison between thyroid malignant and normal groups. *BMI* body mass index; *BP* blood pressure; *LDL-C* low-density lipoprotein cholesterol; *HDL-C* high-density lipoprotein cholesterol; *HbA1c* glycosylated hemoglobin; *FBG* fasting blood glucose; *UA* uric acid; *SAS* self-rating anxiety scale; *SDS* self-rating depression scale; *PSQI* Pittsburgh Sleep Quality Index; *N/A* not available.

### Influence of sleep quality on the risk of thyroid malignant nodule

Thyroid nodule patients were divided into two groups according to the Pittsburgh Sleep Quality Index (PSQI). The poor sleep quality group (n = 280) has a higher incidence of thyroid cancer than good sleep quality group (n = 146), the values were 22.14% *vs* 6.89%, *p* < 0.05. Pearson’s *r* = 0.193, *p* < 0.05.

Next, we used logistic regression analysis to model the association between poor sleep quality and malignant nodule risk, as shown in Table 2. In our sample, the people with poor sleep quality had a higher risk of thyroid malignant nodule relative to those with good sleep quality, after adjusting for sex, age, duration, BMI, BP, UA, thyroid function, SAS and SDS scores, adjusted OR = 1.361, 95% CI 1.122 to 1.651. The contained components sleep latency and daytime dysfunction were also independent risk factors of malignant nodule, adjusted OR = 2.745, 95% CI 1.393 to 5.409, and adjusted OR = 2.685, 95% CI 1.394 to 5.169, respectively.

**Table 2:** Risk of thyroid malignancy according to sleep quality.

	Unadjusted		Adjusted*	
	OR (95% CI)	*P* value	OR (95% CI)	*P* value
Poor sleep quality (> 7)	1.107 (1.033 to 1.186)	0.004	1.361 (1.122 to 1.651)	0.002
Sleep latency score	1.279 (1.005 to 1.628)	0.046	2.745 (1.393 to 5.409)	0.004
Daytime dysfunction score	1.395 (1.093 to 1.780)	0.007	2.685 (1.394 to 5.169)	0.003

*Adjusted for sex, age, duration, BMI, BP, UA, thyroid function, SAS and SDS score. *OR* odds ratio; *CI* confidence interval; *PSQI* Pittsburgh Sleep Quality Index.

Meanwhile, paired t-test showed that the average course of sleep problems was 4.45 ± 6.79 years, and the average course of thyroid nodule diseases was 3.53 ± 4.82 years, the subtracted value (the former minus the latter) was positive 0.91 ± 7.68, 95% CI 0.166 to 1.637, *p* < 0.05. This result suggested sleep disorders might occur before thyroid nodules appeared.

### Immunohistochemistry staining localization of circadian rhythm genes in thyroid tissues

Among the patients with malignant nodules, some were concerned about the operation, some were lost to follow-up, some refused to join the histological study, and some tissues were abandoned because of formalin immersion. Then we totally collected 56 fresh tissues from 40 patients after surgical treatment in our hospital for immunohistochemistry, RT-PCR and Western-Blotting analysis. Malignant nodule group (n = 20), benign nodule group (n = 20), and adjacent normal group (n = 16, 4 adjacent to malignant nodules and 12 adjacent to benign nodules) were involved in the experiments. The pathological types of the malignant nodules included 17 papillary thyroid carcinomas and 3 follicular thyroid carcinomas, and that of most benign nodules were nodular goiter. According to the operation schedule of our hospital, most thyroid tissues were collected at 8 am–10 am (16 malignant nodules and 17 benign nodules), and a small part were collected at 1 pm–2 pm (4 malignant nodules and 3 benign nodules).

**Figure 2:**
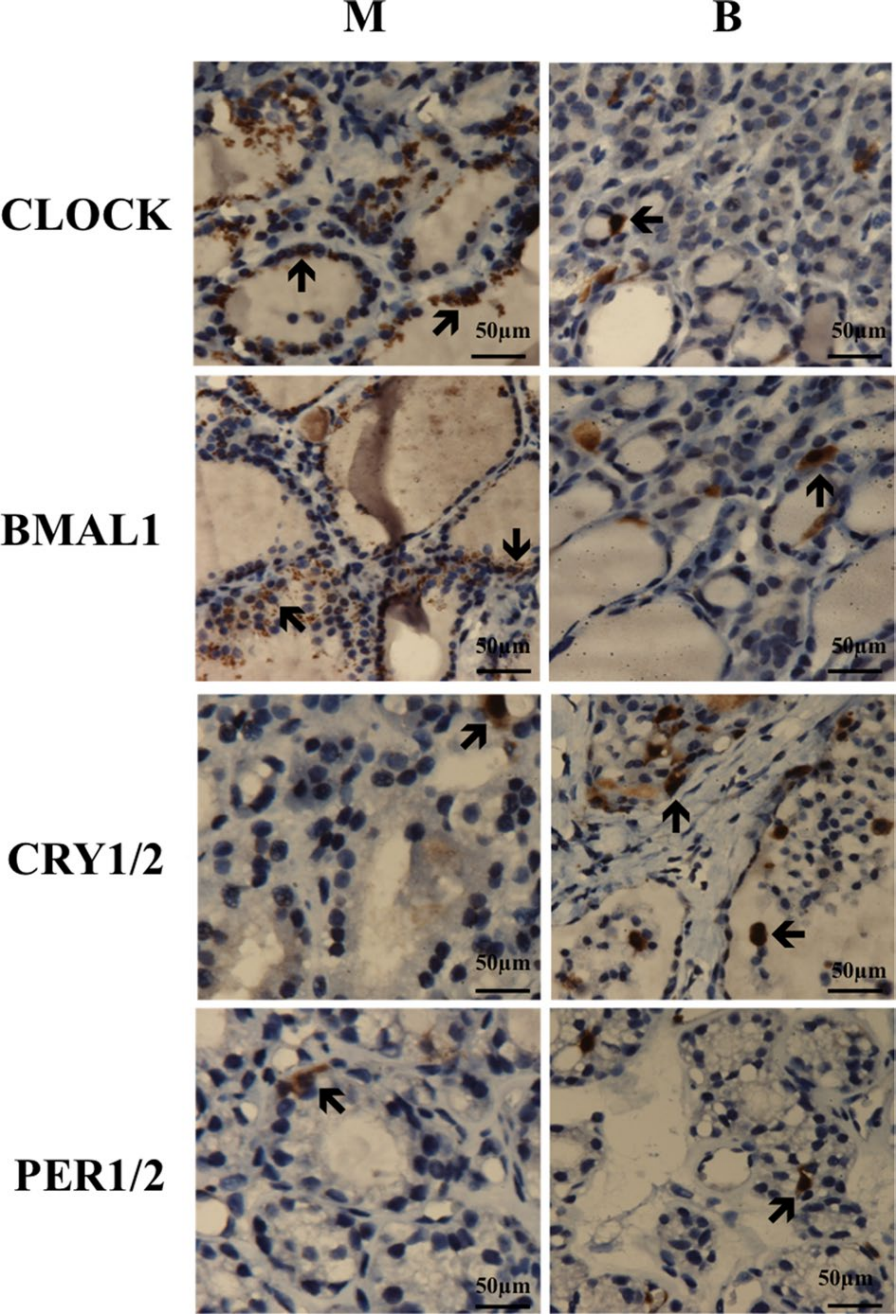
Immunohistochemical staining of CLOCK, BMAL1, CRYs and PERs in thyroid malignant group (n = 8) and benign group (n = 8), normal group (n = 6) was not shown. The typical stained cells were marked with arrows. *M* malignant nodule group; *B* benign nodule group; *N* adjacent normal group.

We can see in Fig. 2, circadian rhythm genes mainly stained in thyroid follicular epithelial cells. CLOCK and BMAL1 were dramatically expressed in thyroid carcinoma but not in benign group, while other genes were in low levels of each group. Besides, CRY1 and CRY2 stained more in benign group than malignant group. No obvious expressions were found in normal group, except for CRY2, data were not shown.

### mRNA expressions of circadian rhythm genes in each group thyroid tissues

**Figure 3:**
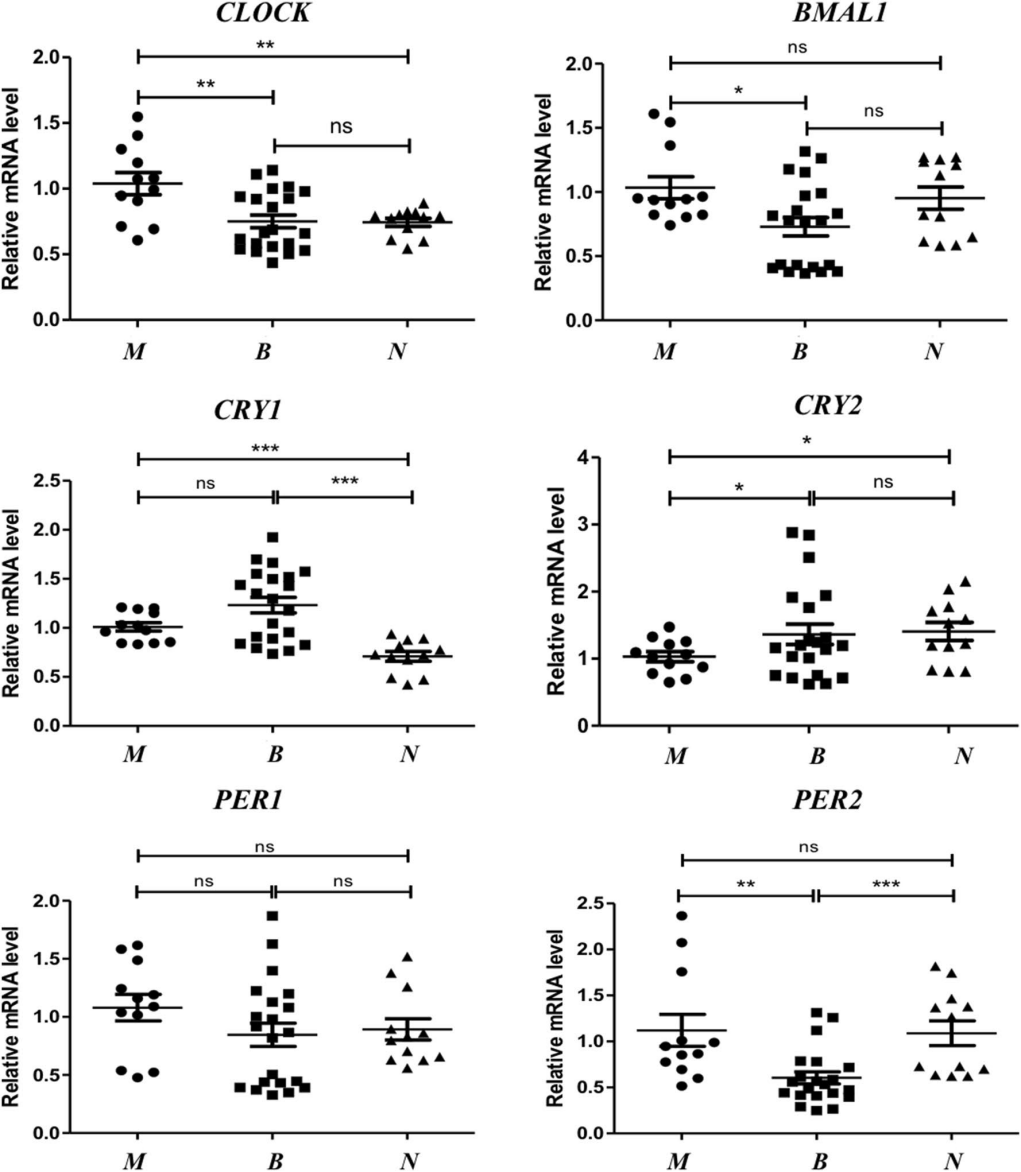
Relative mRNA expression levels of *CLOCK, BMAL1, CRYs* and *PERs* in thyroid malignant group (n = 12), benign group (n = 20) and normal group (n = 12). *p < 0.05, **p < 0.01, ***p < 0.0001. M, malignant nodule group; B, benign nodule group; N, adjacent normal group.

As shown in Fig. 3, *CLOCK* and *BMAL1* mRNA expressed higher in malignant group than in benign and normal groups. Especially for *CLOCK* gene, *p* < 0.01 *vs* the other two groups. As for *BMAL1*, *p* < 0.05 *vs* benign group. The expression of *CRY1* in malignant group was in the middle of the three groups, while that of *CRY2* was relatively in lower levels than benign and normal groups, *p* < 0.05. Finally, *PER1* and *PER2* in the three groups had no practical significance, except the high levels of *PER2* in malignant group, *p* < 0.01 *vs* benign group.

When benign group compared with normal group, there were no statistical significance for *CLOCK、BMAL1、CRY2、PER1*. However, the levels of *CRY1* in benign group increased than normal group, and *PER2* decreased in contrast.

### Protein expressions of circadian rhythm genes in each group thyroid tissues

**Figure 4:**
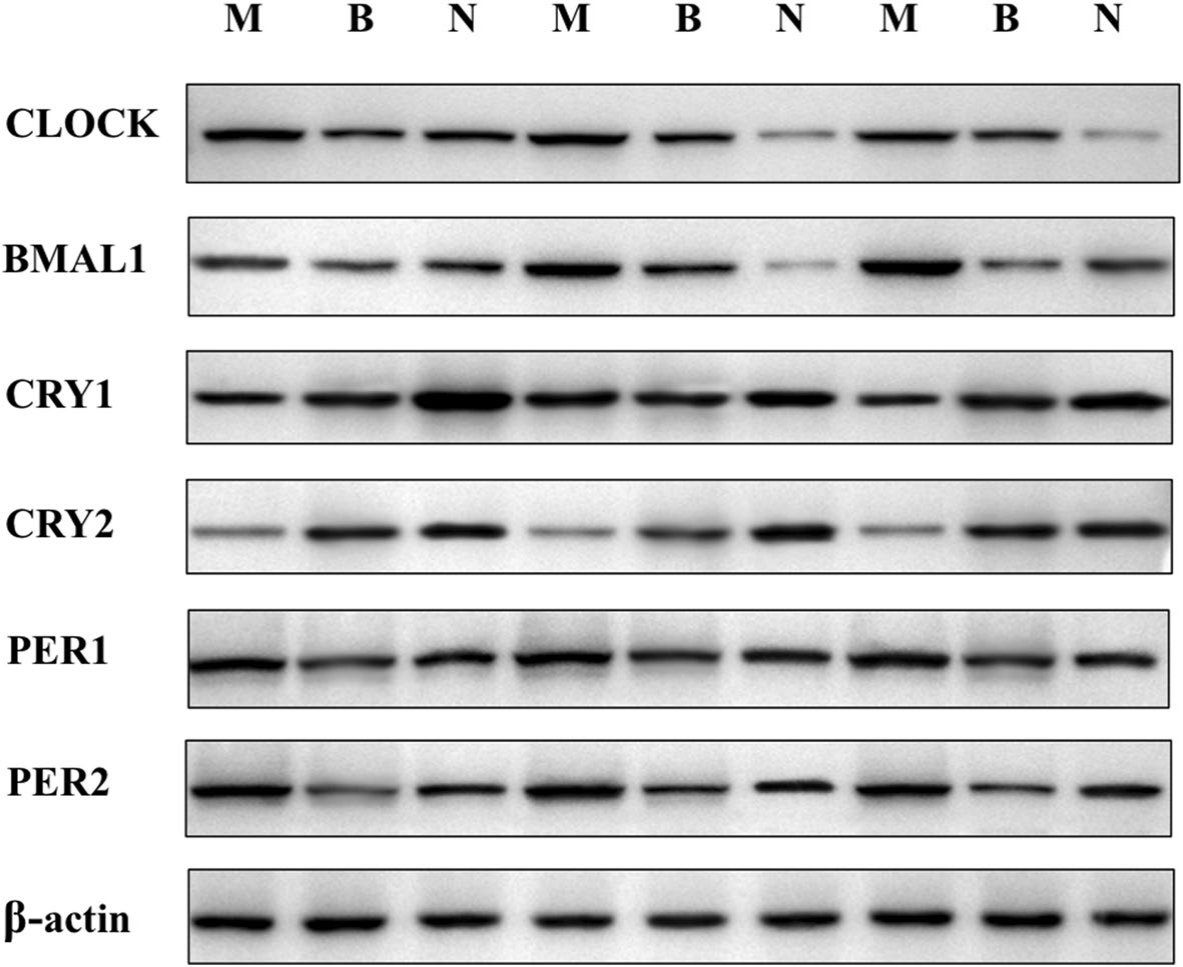
The density of each band was measured by densitometry, and β-actin was used as an internal control. *M* malignant nodule group; *B* benign nodule group; *N* adjacent normal group. Full-length blots/gels were presented in Supplementary File.

**Figure 5:**
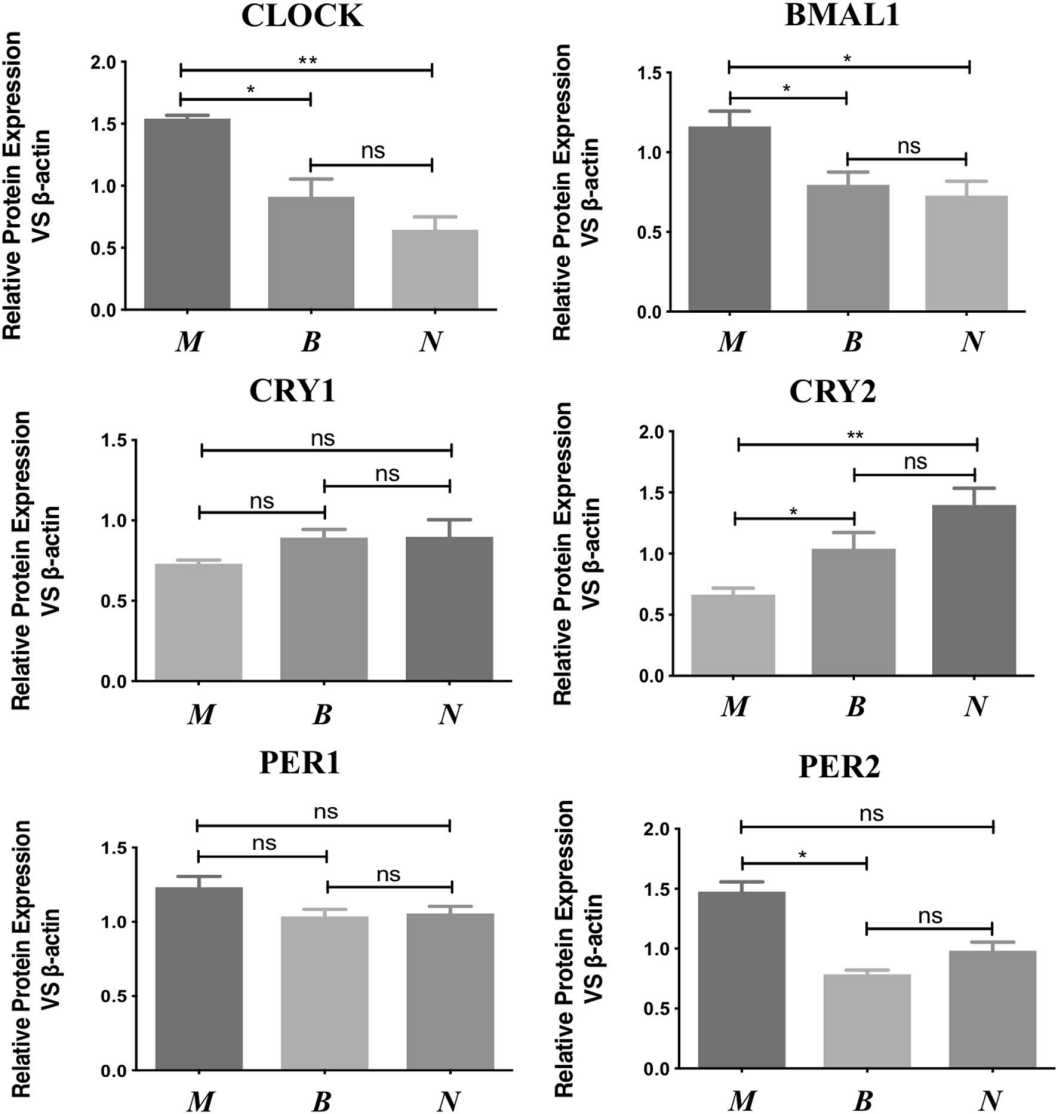
Relative protein expression levels of CLOCK, BMAL1, CRYs and PERs in thyroid malignant group (n = 20), benign group (n = 20) and normal group (n = 16). *p < 0.05, **p < 0.01. M, malignant nodule group; *B* benign nodule group; *N* adjacent normal group.

In Western-blotting analysis, we can see in Figs. 4 and 5, the expressions of CLOCK and BMAL1 in three groups showed a similar trend to mRNA results. Both genes in malignant groups had statistic significance when compared with benign and normal groups, *p* < 0.05. The levels of PER2 was also increased in malignant group, *p* < 0.05 *vs* benign group. However, the expression of CRY2 in the three groups was opposite to that of other genes. The CRY2 protein levels in malignant group obviously expressed lower than the other two groups, *p* < 0.05. All the genes did not make sense in benign and normal groups, and CRY1 and PER1 had no significant difference in the three groups.

Considering the fact that expression levels of circadian genes are varying according to circadian time, we then analyzed individual data and found the expression levels of CLOCK and BAML1 were increased with circadian time while CRY2 and PER2 were decreased (8am to 2 pm). However, there was no significant difference in the collection time between benign and malignant nodules, *p* > 0.05.

### The relationship between sleep quality and the expressions of each gene

We chose CLOCK, BMAL1, CRY2 and PER2 genes, which were significant in the comparison of malignant and benign nodules in both RT-PCR and Western-Blotting analysis. Firstly, we compared protein expression levels of the circadian rhythm genes in good sleep quality group (n = 9) and poor sleep quality group (n = 31). CLOCK、BMAL1 and PER2 levels were increased and CRY2 was decreased in PSQI > 7, especially CLOCK gene, *p* < 0.05.

**Figure 6:**
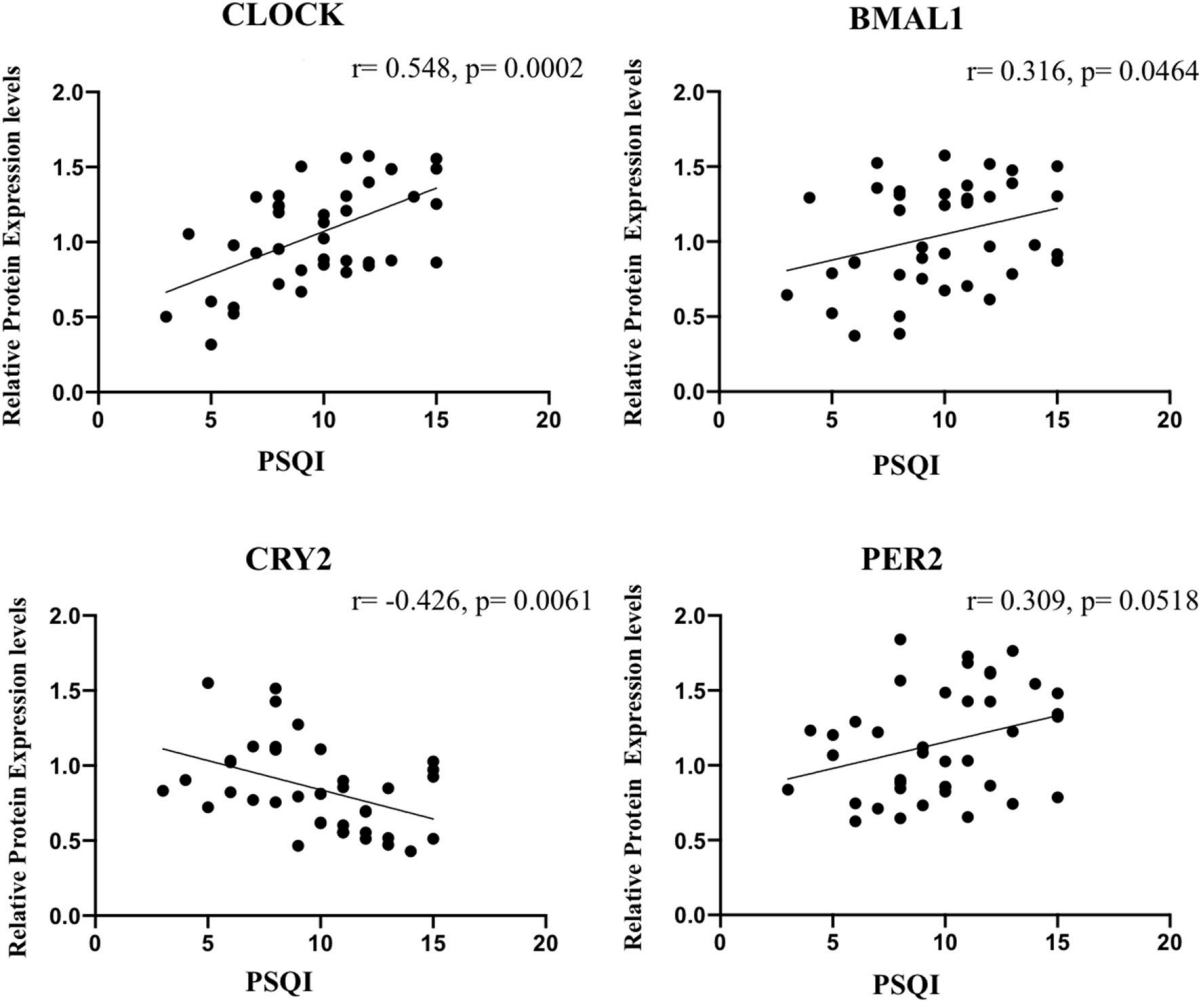
The relationship between PSQI of 40 thyroid nodule patients and the relative protein expression levels of CLOCK, BMAL1, CRY2 and PER2.

Then the correlation was analyzed between PSQI of the 40 patients and the relative protein expression levels of the circadian genes according to the above results, shown in Fig. 6. Among the four circadian rhythm genes tested in our study, the expressions of CLOCK and BMAL1 were significantly positively correlated with PSQI (r = 0.548, *p* = 0.0002; r = 0.316, *p* = 0.0464). While CRY2, was negatively correlated with PSQI (r =  − 0.426, *p* = 0.0061). On the other hand, PER2 made no sense with PSQI (r = 0.309, *p* = 0.0518). In addition, we found that the protein expression levels of these four genes were statistically associated with thyroid malignancy. For CLOCK, the Pearson’s *r* = 0.627, *p* = 0.000. For BMAL1, the Pearson’s *r* = 0.511, *p* = 0.001. For CRY2, the Pearson’s *r* =  − 0.515, *p* = 0.001. For PER2 the Pearson’s *r* = 0.793, *p* = 0.000.

## Discussion

The damage of circadian clock is associated with increased risk of cancer, metabolic syndrome, cardiovascular dysfunction, cognitive impairment, and immune system disorders^22^. In addition, circadian rhythm disorders have been reported to reduce the survival rate of cancer patients^23,24^. The International Agency for Research on Cancer has concluded that “night shift work that involving circadian rhythm damage may carcinogenic to humans”, based on the limited evidence for the carcinogenicity of night shift-work people^25^. Genetic or functional destruction of the molecular clock may lead to genomic instability and accelerate cell proliferation, both of which are conducive to carcinogenesis^26^. Therefore, the abnormal expression of circadian clock genes may have an important effect on the ability of trans activation and apoptosis of downstream cell cycle targets, to some extent can promote carcinogenesis^27^. These molecular rhythms regulate several key aspects of cell and tissue functions, which have far-reaching significance for disease prevention and management as well as public health problem^28^. Thus we put the circadian clocks in a central position, for the molecular understanding of the circadian rhythms has been opening up new research directions and treatment methods for us ^29^.

Recent findings reveal that the circadian rhythms have a fixed time relationship with cell cycles in vivo, which jointly regulate the metabolism and physiology of the body^30–32^. In the endocrine system, hypothalamic-pituitary-thyroid (HPT) axis related hormones secretions are tightly modulated by the circadian system. Moreover, studies have displayed that thyroid cells established from a poorly differentiated thyroid carcinoma showing altered circadian oscillations^33^. At present, the molecular model used to generate circadian oscillations is an interlocking negative feedback loop based on gene expressions. The positive feedback loops mainly include CLOCK, BMAL1 and NPAS2, while PERs and CRYs are the components of negative feedback regulations^34,35^. We then chose CLOCK, BMAL1, CRY1, CRY2, PER1 and PER2 to observe the changes of circadian rhythm genes in thyroid malignant tissues. We found that the mRNA expression of *CLOCK* and *BMAL1* in thyroid carcinoma tissues (PTC) was significantly higher than that in benign and normal tissues, while the expression of *CRY2* was decreased (Fig. 3), indicating that circadian biological rhythm was involved in the occurrence of the disease. In the immunohistochemical experiment, the CLOCK and BMAL1 were dramatically stained in thyroid carcinoma but not in benign groups, while other genes were in low levels (Fig. 2). Further analysis by quantitation western-blot, the expression trend of each gene was consistent with that of mRNA (Figs. 4 and 5). When benign groups compared with normal groups, no significant difference was detected in protein levels, which provided the evidence that circadian rhythm genes mainly altered in malignant nodules.

Some studies showed that the growth and proliferation of mouse cells with CLOCK gene mutation were inhibited, which may be related to the down-regulation of cell cycles proliferation genes and up-regulation of cell cycles suppressor genes^36^. The other was loss of the trans acting factors of CLOCK gene in the negative feedback loop, thus the negative regulation of transcription was weakened, which destroyed the normal loop of biological rhythm genes and affected the normal operation of cell cycles^37^. Mannic demonstrated for the first time that core-clock gene expression levels were altered in thyroid malignancies, namely the up-regulation of BMAL1 and down-regulation of CRY2 in FTCs and PTCs^37^. In line with the previous studies, we explored the changes of circadian rhythm genes of elderly thyroid nodules from the protein expression levels, making the results more convinced. We investigated human thyroid circadian genes expression and its potential role in thyroid tumor progression, contributing to the unresolved issue of the malignant nodule preoperative diagnosis, yet the regulatory mechanism needed to be further clarified.

In modern society, the damage of circadian rhythms leads to poor sleep and further interruption of sleep–wake cycles^28^. Meanwhile, the circadian rhythm and the sleep/wake state are the two processes that link sleep quality with organ dysfunctions. A short sleep duration was associated with subclinical thyroid diseases, hypertension and type 2 diabetes^38–40^, and a long sleep duration had a direct relationship with increased mortality^41^. Other studies showed that Per3 gene polymorphism combined with short sleep duration, which affected transient emotional states in women^42^. In adult HIV patients, circadian rhythm genes polymorphisms were related to sleep disruption, sleep duration, and circadian phases^43^. All these implied that sleep deprivation and circadian rhythm disorders, such as shift work and social jet lag, were well proven to interfere with many aspects of health and to cause serious diseases, including metabolic syndrome, psychiatric illness and malignant transformation^8,44^.

In our study, we collected clinical data of 354 benign thyroid nodule patients, 72 malignant ones and 53 healthy people, all the participants were elderly. Of all the hormones, TSH is best known to be primarily influenced by the circadian rhythms^9,45,46^. However, TSH levels had no difference in the three groups in our results, instead, TGAB, TPOAB and TRAB had changed (Table 1). In can be explained that thyroid functions of the people enrolled were completely normal, and autoimmunity may be related to the development of thyroid malignancy. By statistical calculation we concluded that poor sleep quality, sleep latency and daytime dysfunction were the independent risk factors of thyroid malignant nodules after adjusted by other impacts (Table 2). When divided into poor and good sleep quality groups, thyroid cancer rate was dramatically increased in PSQI > 7. Moreover, of the 40 patients, CLOCK, BMAL1 and PER2 expression levels were increased and CRY2 was decreased in those who with poor sleep quality. We then analyzed the relationship between the protein expression levels of circadian genes in 40 thyroid tissues and the PSQI of corresponding patients. We found CLOCK, BMAL1 levels were positively correlated with PSQI, while CRY2 was negatively correlated (Fig. 6). Interestingly, these circadian genes were associated with thyroid malignancy in the mean time, and the correlation directions were consistent with that of PSQI. In other words, the worse the sleep quality the higher expression levels of CLOCK and BMAL1, and the lower level of CRY2, which also mean the greater risks of thyroid malignancy. The key point was that the average course of sleep problems was 4.45 ± 6.79 years, and that of thyroid nodule diseases was 3.53 ± 4.82 years, the subtracted value was positive, indicating sleep disorders may be involved in the occurrence of elderly thyroid malignancy through the high expressions of CLOCK and BMAL1, and low expression of CRY2. Coincidentally, a recent large US cohort study of 460,000 participants showed that exposure to artificial light at night (LAN) significantly increased the risk of thyroid cancer^47^, which further proved our results.

In conclusion, our study combined circadian rhythm genes of thyroid malignancy with sleep disorders to form a closed loop research, as well as joined basic experiments with clinical data. We also explored the sleep effects on thyroid malignancy, the results suggested regular lifestyle and good sleep quality may give rise to low risks of thyroid cancer in elderly people, and provided new clues for clinical evaluation and intervention.

## Supplementary Information

Supplementary Information.
