# Supplementary material for: Alterations of sleep quality and circadian rhythm genes expression in elderly thyroid nodule patients and risks associated with thyroid malignancy
Source: Sci Rep. 2021 Jul 1;11:13682. doi: 10.1038/s41598-021-93106-x (PMC8249375; doi:10.1038/s41598-021-93106-x)
Supplement: Supplementary file 1 — Supplementary Information. [file 41598_2021_93106_MOESM1_ESM.pdf]

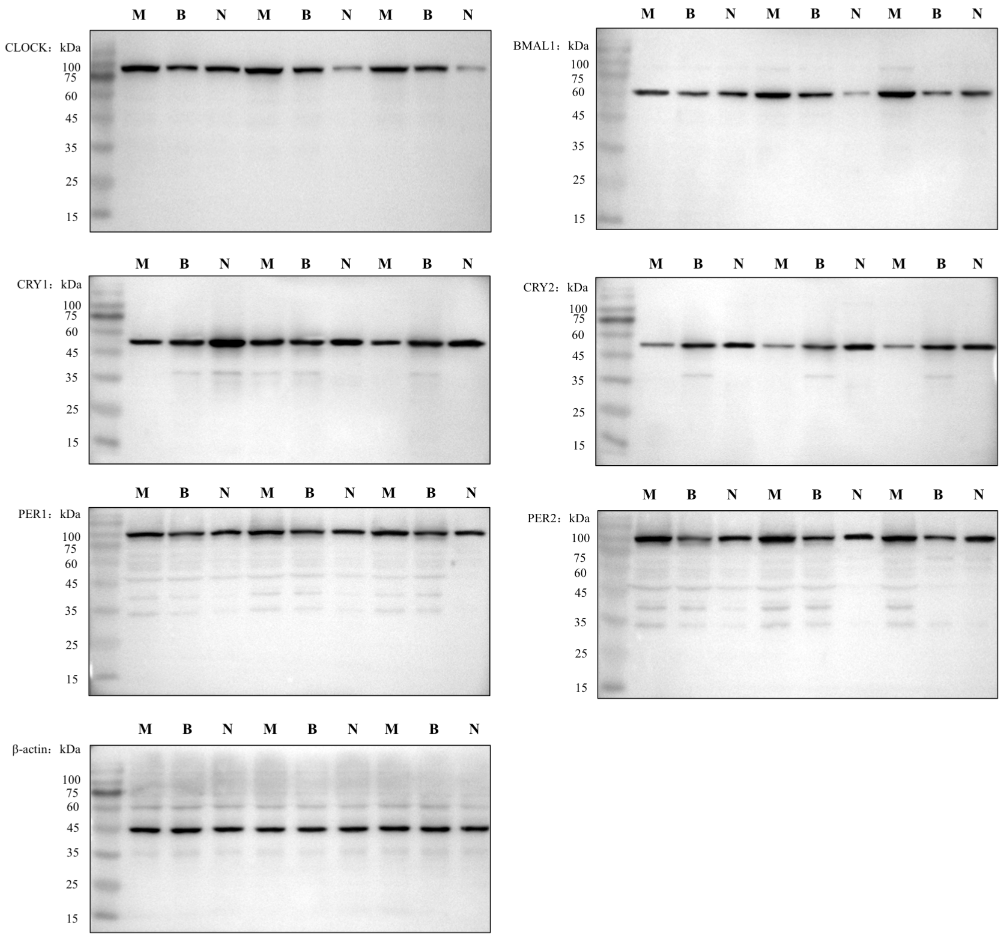

Figure 4. The density of each band was measured by densitometry, and β-actin was used as an internal control. M, malignant nodule group; B, benign nodule group; N, adjacent normal group.

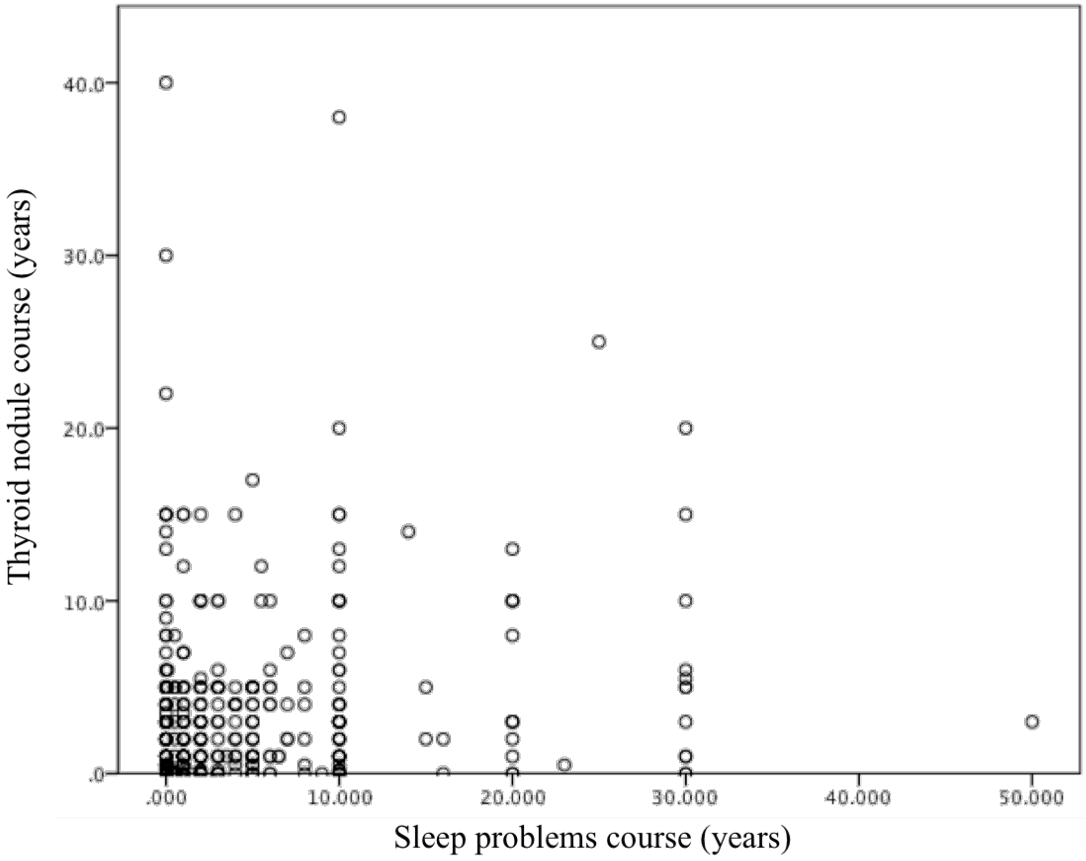

Supplementary figure 1. Scatter plot of individual data (the course of thyroid nodule diseases *vs* sleep problems).

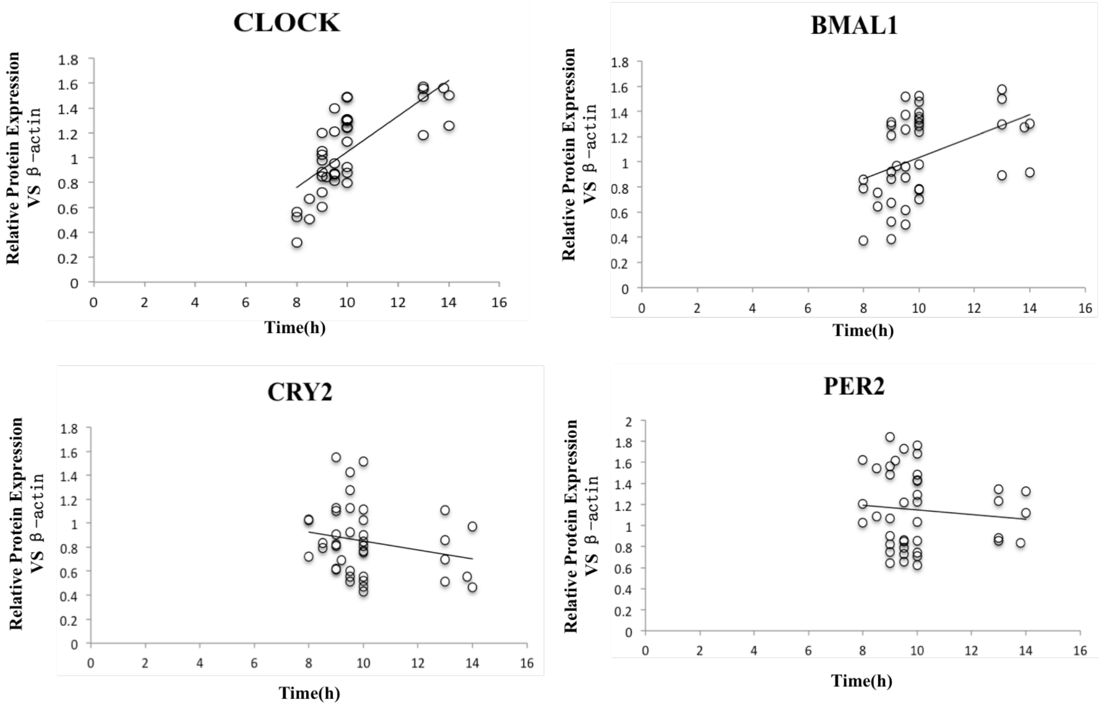

Supplementary figure 2. Scatter plots of genes relative protein expression levels by circadian time (from 8am to 2pm).
